# New Insights into the Synergistic Bioactivities of *Zingiber officinale* (Rosc.) and *Humulus lupulus* (L.) Essential Oils: Targeting Tyrosinase Inhibition and Antioxidant Mechanisms

**DOI:** 10.3390/molecules30153294

**Published:** 2025-08-06

**Authors:** Hubert Sytykiewicz, Sylwia Goławska, Iwona Łukasik

**Affiliations:** Faculty of Natural Sciences, Institute of Biological Sciences, University of Siedlce, 14 Prusa St., 08-110 Siedlce, Poland; sylwia.golawska@uws.edu.pl (S.G.); iwona.lukasik@uws.edu.pl (I.Ł.)

**Keywords:** essential oils, anti-tyrosinase capacity, lipid peroxidation inhibition, radical scavenging, *Zingiber officinale* rhizome, *Humulus lupulus* strobilus

## Abstract

Essential oils (EOs) constitute intricate mixtures of volatile phytochemicals that have garnered significant attention due to their multifaceted biological effects. Notably, the presence of bioactive constituents capable of inhibiting tyrosinase enzyme activity and scavenging reactive oxygen species (ROS) underpins their potential utility in skin-related applications, particularly through the modulation of melanin biosynthesis and protection of skin-relevant cells from oxidative damage—a primary contributor to hyperpigmentation disorders. *Zingiber officinale* Rosc. (ginger) and *Humulus lupulus* L. (hop) are medicinal plants widely recognized for their diverse pharmacological properties. To the best of our knowledge, this study provides the first report on the synergistic interactions between essential oils derived from these species (referred to as EOZ and EOH) offering novel insights into their combined bioactivity. The purpose of this study was to evaluate essential oils extracted from ginger rhizomes and hop strobiles with respect to the following: (1) chemical composition, determined by gas chromatography–mass spectrometry (GC-MS); (2) tyrosinase inhibitory activity; (3) capacity to inhibit linoleic acid peroxidation; (4) ABTS^•+^ radical scavenging potential. Furthermore, the study utilizes both the combination index (CI) and dose reduction index (DRI) as quantitative parameters to evaluate the nature of interactions and the dose-sparing efficacy of essential oil (EO) combinations. GC–MS analysis identified EOZ as a zingiberene-rich chemotype, containing abundant sesquiterpene hydrocarbons such as *α*-zingiberene, *β*-bisabolene, and *α*-curcumene, while EOH exhibited a caryophyllene diol/cubenol-type profile, dominated by oxygenated sesquiterpenes including *β*-caryophyllene-9,10-diol and 1-*epi*-cubenol. In vitro tests demonstrated that both oils, individually and in combination, showed notable anti-tyrosinase, radical scavenging, and lipid peroxidation inhibitory effects. These results support their multifunctional bioactivity profiles with possible relevance to skin care formulations, warranting further investigation.

## 1. Introduction

*Zingiber officinale* Roscoe (family Zingiberaceae) is an herbaceous perennial native to Southeast Asia, widely cultivated for its aromatic underground rhizomes rich in bioactive compounds such as gingerols and shogaols [1,2,3]. *Humulus lupulus* L. (family Cannabaceae) is a dioecious, climbing perennial plant producing female strobiles characterized by bitter *α*- and *β*-acids, essential oils including humulene and caryophyllene, and prenylated flavonoids such as xanthohumol [4]. The unique phytochemical composition of these plants warrants comprehensive investigation of their essential oil fractions in the context of their potential dermatological and health-promoting applications.

Oxidative stress, defined as an imbalance between the production of reactive oxygen species (ROS) and endogenous antioxidant defenses, is a pathogenic factor implicated in a variety of chronic diseases and skin-related disorders, including hyperpigmentation, photoaging, and inflammatory conditions [5,6,7]. In the skin, ROS contribute to cellular dysfunction and tissue impairment, accelerating aging processes and exacerbating pigmentation disorders [8]. Lipid peroxidation of polyunsaturated fatty acids such as linoleic acid represents a critical pathway of oxidative damage, leading to membrane disruption and generation of cytotoxic products [8]. Essential oils (EOs)—complex mixtures of volatile phytochemicals such as terpenes, phenolics, and aldehydes—are considered potential sources of antioxidant compounds [9,10,11]. However, to date, only a limited number of EOs have shown inhibitory activity against tyrosinase, a key enzyme in melanin biosynthesis, indicating their potential to reduce hyperpigmentation [12,13]. This effect may be mediated by phytochemicals that chelate the copper ions of tyrosinase or mitigate oxidative stress, which in turn stimulates melanin production [14,15].

While the rhizomes of *Zingiber officinale* and the strobiles of *Humulus lupulus* are well-characterized sources of bioactive compounds with established pharmacological properties, their essential oils remain underexplored, especially regarding their whole-oil biological activity profiles pertinent to skin health. Most research has focused on isolated constituents or non-volatile extracts rather than the complete essential oil fractions [2,4]. Given the increasing emphasis on natural and multifunctional agents in dermatological research, essential oils with demonstrated antioxidant, antimelanogenic, and anti-inflammatory properties have attracted significant attention. Such bioactive formulations, which combine multiple protective mechanisms while minimizing synthetic components, represent promising candidates for advanced cosmeceutical and dermatological applications. Despite the growing interest in natural antioxidants and tyrosinase inhibitors, no previous studies have examined the combinatory effects of EOZ and EOH in this context. This study is the first to report on the synergistic bioactivities of these two essential oils, introducing an advanced framework for evaluating their efficacy using combination index (CI) and dose reduction index (DRI) analyses. These parameters offer valuable insights into the dose-sparing and potentiation effects of binary mixtures of the essential oils. This research contributes novel insights by demonstrating the enhanced efficacy of EOZ and EOH when applied in combination—supporting a scientifically substantiated strategy for the development of multifunctional, plant-based cosmeceuticals.

Accordingly, the present study aimed to investigate the following: (1) the chemical composition of essential oils extracted from the rhizomes of *Zingiber officinale* (EOZ) and the strobiles of *Humulus lupulus* (EOH), as determined by gas chromatography–mass spectrometry (GC-MS); (2) their inhibitory effects on tyrosinase activity, serving as an in vitro model for antimelanogenic potential; (3) their antioxidant capacity, evaluated via linoleic acid peroxidation inhibition and ABTS^•+^ radical scavenging assays. Furthermore, the study assessed the nature of interactions between EOZ and EOH when combined, to determine whether their joint effects on these bioactivities are synergistic, additive, or antagonistic.

## 2. Results and Discussion

### 2.1. Chemical Composition of the Tested Essential Oils

The essential oil extracted from the rhizomes of *Zingiber officinale* Rosc. (EOZ) exhibits a chemically rich and structurally diverse volatile profile, primarily composed of sesquiterpene hydrocarbons (Table 1, Figure S1). Gas chromatography–mass spectrometry (GC-MS) analysis identified 71 distinct volatile constituents, accounting for 89.3% of the total volatile composition. This profile is representative of a zingiberene-rich chemotype, characterized by the high abundance of *α*-zingiberene (11.4%), along with *β*-bisabolene (8.9%) and *α*-curcumene (7.8%) [16,17]. These major sesquiterpenes form the aromatic backbone of the oil and are responsible for much of its bioactivity, including anti-inflammatory and antioxidant potential. Monoterpenes, although present in lower concentrations, significantly influence the volatile scent profile and therapeutic properties [16,18]. Notably, oxygenated monoterpenes such as geranial (12.7%) and neral (7.2%)—known collectively as citral isomers—impart fresh, citrus-like olfactory notes and are associated with documented antimicrobial, antifungal, and anti-inflammatory effects [18,19]. Despite their prominence, citral isomers act more as functional modifiers than as chemotaxonomic markers. Additional monoterpenes, including camphene (6.8%), *β*-phellandrene (5.7%), and 1,8-cineole (4.8%), contribute camphoraceous and herbal aromas, enhancing the oil’s sensory and pharmacological complexity [20]. Minor oxygenated sesquiterpenes and alcohols—borneol, *α*-terpineol, and several eudesmol isomers—though present in trace amounts, may exert synergistic or modulatory effects, enhancing the bioactivity of major constituents [16,19]. This chemical pattern reflects a biosynthetic orientation toward sesquiterpene dominance in mature ginger rhizomes, shaped by genetic, environmental, and post-harvest factors [21]. The clear predominance of *α*-zingiberene confirms the zingiberene-rich chemotype—a classification crucial for ensuring quality control and therapeutic consistency in pharmaceutical and commercial applications of ginger oil [19]. While oxygenated monoterpenes such as citral enhance functional value, they do not override the sesquiterpene-centric chemotaxonomic identity. This composition corresponds to mature, fully developed rhizomes, where terpene biosynthesis is geared toward sesquiterpene accumulation and diversification.

In contrast, the essential oil isolated from the strobiles of *Humulus lupulus* L. (EOH) displays a distinct chemotaxonomic signature, dominated by oxygenated sesquiterpenes rather than hydrocarbons (Table 2, Figure S2). GC-MS analysis revealed 36 distinct volatile compounds, representing 71.6% of the total volatile profile. This EO may be classified as a caryophyllene diol/cubenol-type chemotype, characterized by the presence of *β*-caryophyllene-9,10-diol isomer 2 (12.7%), 1-*epi*-cubenol (12.2%), *trans*-anethole (7.5%), *γ*-cadinene (4.8%), *trans*-calamenene (4.3%), and *γ*-muurolene (3.5%). These components are consistent with oxidative terpene metabolism in fully developed strobiles [22,23]. Key indicators of oxidative biotransformation, such as 1,2-humulenepoxide (2.3%), *trans*-caryophyllene oxide (2.2%), and cadalene (2.1%), suggest post-harvest aging or natural enzymatic modification of sesquiterpene hydrocarbons [24,25]. The minimal content of monoterpenes—*α*-pinene (0.5%), limonene (0.2%), and linalool (0.5%)—is characteristic of aged hop material, where monoterpenes have likely evaporated or degraded during drying and storage [25,26]. Significant levels of aliphatic ketones, such as methyl isobutyl ketone (1.8%) and 2-undecanone (2.1%), further support oxidative degradation of fatty acids or microbially mediated transformations during prolonged storage [27,28,29]. Within this chemically diverse profile, one particularly intense signal warrants thorough assessment. A prominent peak observed at a retention time (RT) of 35.15 min exhibited notably high intensity in the total ion chromatogram (TIC). Detailed analysis of the corresponding mass spectrum revealed the presence of fragment ions attributable to multiple structurally related sesquiterpene alcohols. Although cubebol was initially considered the primary compound eluting at this RT, the spectral complexity and the known similarity in retention indices of compounds such as 4-*epi*-cubebol, 1-*epi*-cubenol, and cadinol isomers suggest a likely co-elution. The overlapping of these components may account for the unusually high peak area observed. Such co-elution phenomena are well-documented in GC-MS analyses of essential oils, particularly when using non-polar columns, where the separation of terpenoid isomers remains challenging. To resolve the potential co-elution of compounds, future studies should employ spectral deconvolution techniques and two-dimensional gas chromatography (GC × GC) for improved resolution and unambiguous identification of individual constituents. Additionally, highly specialized analytical approaches, such as high-resolution mass spectrometry (HRMS) or nuclear magnetic resonance (NMR) spectroscopy, may be necessary to definitively characterize the compounds involved. The chemical complexity of EOH is strongly influenced by varietal differences, harvest timing, climate, soil characteristics, and post-harvest treatment [23,25]. Early-harvested strobiles are rich in *β*-myrcene, a monoterpene hydrocarbon lost in mature stages. Later harvests yield oils dominated by oxygenated sesquiterpenes due to ongoing enzymatic transformations [27,28,29]. Drying conditions and storage further shape the volatile profile—monoterpenes are prone to oxidation, while more stable sesquiterpenes accumulate over time [26,30]. The method of essential oil extraction is another critical variable. Hydrodistillation may lead to thermal degradation of labile components, whereas supercritical CO_2_ extraction better preserves thermosensitive and low-abundance constituents [30]. This highlights the need for standardized extraction and analytical procedures, especially in chemotaxonomic classification, pharmaceutical development, and regulatory compliance. From a taxonomic and quality-control perspective, identification of dominant markers such as caryophyllene oxide, 1-*epi*-cubenol, *epi*-*α*-cadinol, and *α*-cadinol provides a robust framework for distinguishing *H. lupulus* from other Cannabaceae species. The current compositional pattern is characteristic of oxidatively mature strobiles, harvested late in development and subject to natural or storage-induced chemical evolution. This EO’s chemotype stands in contrast to the myrcene-dominant types typically used in brewing, where *α*-humulene and *β*-myrcene dominate the profile [31,32]. Here, the low content of *α*-humulene (0.8%) and absence of *β*-myrcene confirms a non-brewing, pharmacological chemotype, suitable for aromatherapeutic and phytopharmaceutical use. Collectively, this fingerprint supports applications in botanical standardization, medicinal plant authentication, and bioactivity prediction models, positioning this essential oil for advanced integrative uses in herbal medicine, cosmeceuticals, and functional product design.

### 2.2. Anti-Tyrosinase Efficacy of the Examined Essential Oils Isolated from Ginger Rhizomes and Hop Strobiles

**Figure 4:**
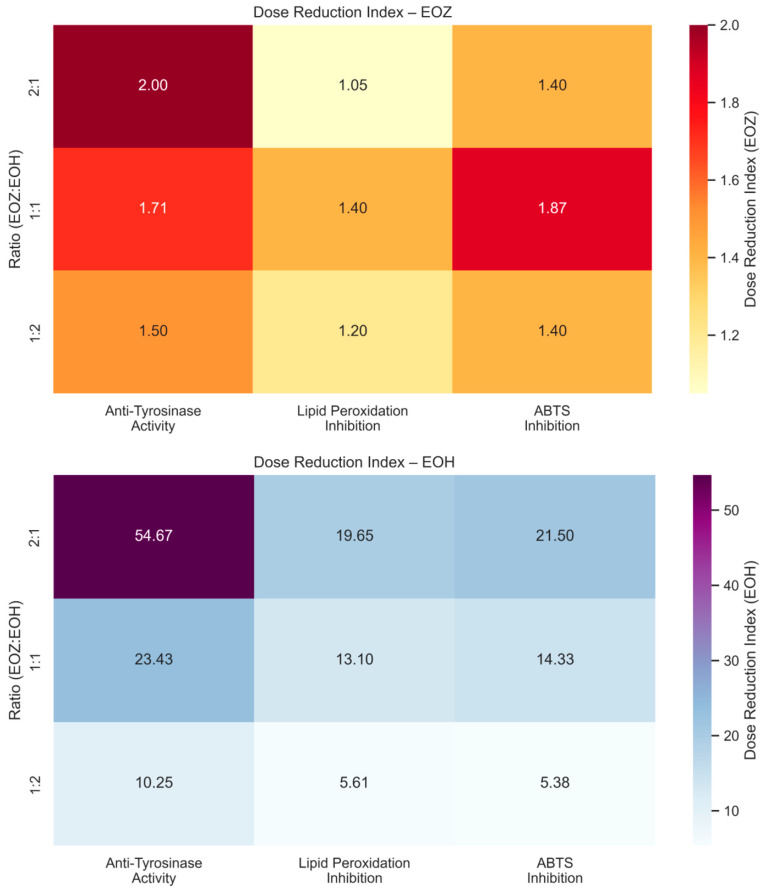
Heatmaps showing dose reduction index (DRI) values for binary mixtures of EOZ (**top**) and EOH (**bottom**) essential oils evaluated in three non-cellular in vitro assays: anti-tyrosinase activity, lipid peroxidation inhibition, and ABTS^•+^ radical scavenging. EOZ and EOH represent essential oils isolated from *Zingiber officinale* (Rosc.) rhizomes and *Humulus lupulus* (L.) strobiles, respectively. Mixtures were prepared at volume ratios of EOZ to EOH (*v*/*v*): 1:1, 1:2, and 2:1. DRI values were calculated using the Chou–Talalay method [33,44,45] as the ratio of the concentration of a single essential oil (Dx) to the concentration in combination (D), according to the following formula: DRI = Dx/D. Interpretation: DRI > 1 indicates a favorable dose reduction achieved by combining EOZ and EOH, meaning that a lower concentration of the individual oil is required in the mixture to elicit the same effect as when used alone. The extent of reduction is expressed as *n*-fold (e.g., DRI = 2 corresponds to a two-fold dose reduction). DRI = 1 reflects no dose reduction (i.e., additive effect), while DRI < 1 suggests a less effective combination.

**Figure 1:**
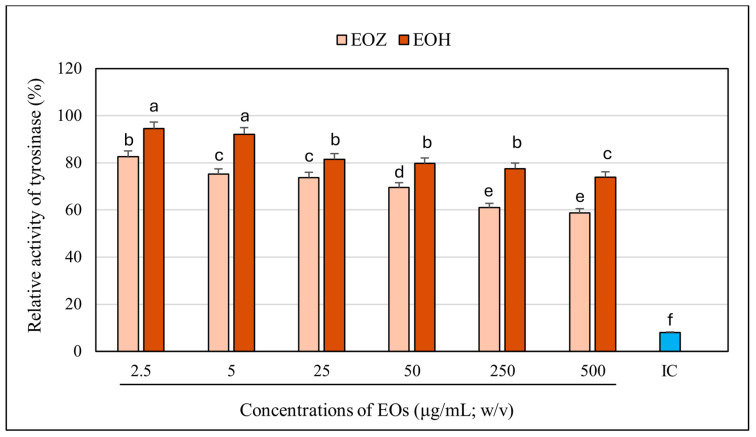
Influence of examined concentrations of the individual essential oils on tyrosinase activity. EOZ and EOH refer to essential oils derived from *Zingiber officinale* (Rosc.) rhizomes and *Humulus lupulus* (L.) strobiles, respectively. Enzyme activity measured in the absence of essential oils was defined as 100%. IC (inhibitor positive control)—kojic acid at a concentration of 710 μg/mL (*w*/*v*) (depicted in blue). Different letters above the error bars represent statistically significant differences in mean enzyme activity values (±SD), determined using Tukey’s test (*p* < 0.05).

**Figure 2:**
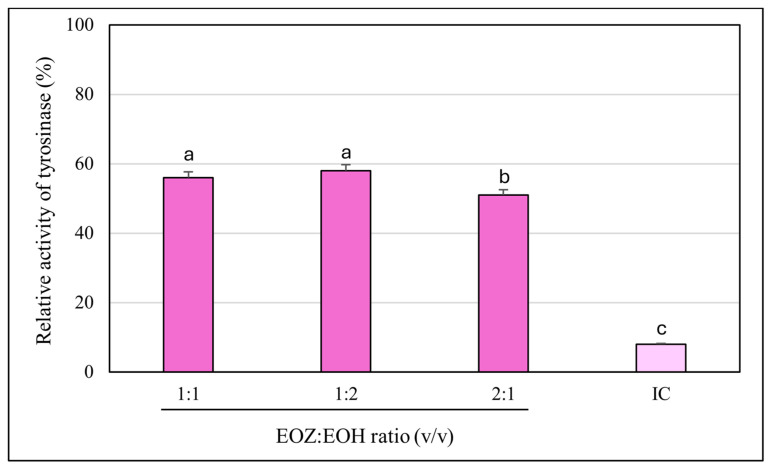
Anti-tyrosinase activity of binary mixtures of essential oils isolated from the rhizomes of *Zingiber officinale* Rosc. (EOZ) and the strobiles of *Humulus lupulus* L. (EOH). Three mixtures were formulated using volumetric ratios (*v*/*v*) of 1:1, 1:2, and 2:1 (EOZ:EOH), at a total essential oil concentration of 250 µg/mL (*w*/*v*), corresponding to individual concentrations of 125:125, 83:167, and 167:83 µg/mL, respectively. Enzyme activity measured in the absence of essential oils was defined as 100%. IC (inhibitor positive control)—kojic acid at a concentration of 710 μg/mL (*w*/*v*). Bars representing the EOZ:EOH mixtures are shown in violet, while the bar for the positive control (kojic acid) is depicted in light violet. Different letters above the error bars represent statistically significant differences in mean enzyme activity values (± SD), based on Tukey’s test (*p* < 0.05).

**Figure 3:**
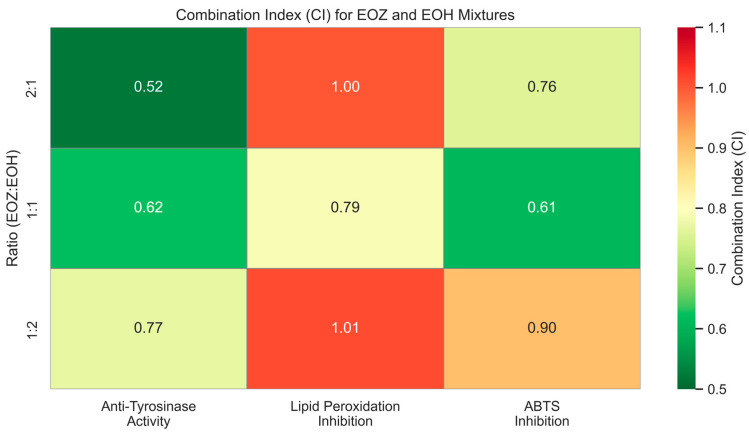
Heatmap illustrating combination index (CI) values for binary mixtures of EOZ and EOH essential oils evaluated in three non-cellular in vitro assays: anti-tyrosinase activity, lipid peroxidation inhibition, and ABTS^•+^ radical scavenging. EOZ and EOH refer to essential oils isolated from *Zingiber officinale* (Rosc.) rhizomes and *Humulus lupulus* (L.) strobiles, respectively. Mixtures were prepared at volume ratios of EOZ to EOH (*v*/*v*): 1:1, 1:2, and 2:1. CI values were calculated based on the median-effect principle using the Chou–Talalay method [33]. Interpretation: CI < 1 indicates synergism, CI ≈ 1 additive effect, and CI > 1 antagonism.

The inhibitory potential of ginger essential oil (EOZ) and hop essential oil (EOH) against tyrosinase was quantitatively evaluated at concentrations of 2.5, 5, 25, 50, 250, and 500 µg/mL (Figure 1). Both oils demonstrated a clear, concentration-dependent pattern of inhibition, although there were marked differences in potency. EOZ exhibited moderate but consistent tyrosinase inhibitory activity, with inhibition rates ranging from 17.4% at 2.5 µg/mL to 41.2% at 500 µg/mL. The gradual increase in activity across the tested range suggests a modest yet progressive effect, particularly at higher concentrations. Conversely, EOH showed comparatively weaker inhibition, increasing from 5.5% to 26.0%, indicating lower efficacy across all concentrations. Notably, EOZ outperformed EOH at every tested level, highlighting its superior intrinsic anti-tyrosinase potential. Due to the lack of 50% inhibition of tyrosinase activity by the tested essential oils, the half-maximal inhibitory concentration (IC_50_) could not be determined. Instead, the IC_20_ (concentration required for 20% inhibition) for each essential oil was calculated to compare their inhibitory potential against tyrosinase (IC_20_ = 3.0 ± 0.12 µg/mL for EOZ and 41.0 ± 1.8 µg/mL for EOH). To further investigate potential synergistic interactions, EOZ and EOH were combined in fixed volume ratios of 1:1, 1:2, and 2:1, at a total concentration of 250 µg/mL. The corresponding inhibition percentages were 44%, 42%, and 49%, respectively (Figure 2), all of which exceeded the activity of the individual oils alone. This enhancement indicates possible interactions that contribute to greater enzyme inhibition. To characterize these interactions, the combination index (CI) was calculated using the Chou–Talalay method [33]. According to standard interpretation, CI values < 1 suggest synergy, CI = 1 indicates additive effects, and CI > 1 reflects antagonism. All tested combinations yielded synergistic effects, with the 2:1 mixture (EOZ:EOH) exhibiting the most pronounced synergy (CI = 0.52 ± 0.01) (Table S1, Figure 3). The 1:1 and 1:2 mixtures also demonstrated synergism, with CI values of 0.62 ± 0.02 and 0.77 ± 0.05, respectively. As a positive control, kojic acid (a reference tyrosinase inhibitor commonly used in dermatological formulations) was tested at a concentration of 710 µg/mL (*w*/*v*), resulting in 92% inhibition of tyrosinase activity (Figure 1 and Figure 2). This benchmark highlights the moderate yet potentially valuable tyrosinase-inhibitory effects of EOZ, EOH, and their synergistic combinations. To quantify the practical implications of synergy, dose reduction index (DRI) values were calculated for each oil in the mixtures (Table S2, Figure 4). DRI values for EOZ ranged approximately from 1.5 to 2.0, indicating a 1.5- to 2-fold reduction in the amount of EOZ required when combined with EOH to achieve the same anti-tyrosinase effect. Remarkably, DRI values for EOH were much higher, ranging from about 10 to over 50, reflecting a significant dose reduction in EOH in mixtures with EOZ. This suggests that much lower concentrations of EOH are needed when combined with EOZ to elicit equivalent enzyme inhibition, highlighting the strong dose-sparing beneficial effects of these mixtures. The results clearly demonstrate that EOZ is more effective than EOH in inhibiting tyrosinase activity. Moreover, the observed synergistic interactions in combined formulations suggest enhanced bioactivity when EOZ and EOH are used in specific ratios. Such synergy may be particularly relevant for the development of multifunctional cosmetic or dermatological agents aimed at modulating melanin biosynthesis and treating or preventing hyperpigmentation disorders.

Tyrosinase (EC 1.14.18.1) is a copper-containing oxidase enzyme that catalyzes two critical steps in melanogenesis: the hydroxylation of L-tyrosine to L-DOPA and the subsequent oxidation of L-DOPA to dopaquinone. Its catalytic activity is central to melanin biosynthesis, making tyrosinase inhibition a primary target for therapeutic and cosmetic interventions aimed at reducing hyperpigmentation [34,35]. Essential oils constitute complex phytochemical matrices predominantly composed of volatile, lipophilic secondary metabolites, with qualitative and quantitative profiles that vary substantially according to plant species, geographic origin, and extraction methods. This chemical heterogeneity often translates into diverse biological activities, including enzyme inhibition [36,37]. The exploration of essential oils with distinctive or under-characterized chemical compositions is warranted, as such profiles may harbor novel bioactive constituents capable of modulating melanogenesis through mechanisms not yet fully elucidated. Furthermore, the combination of essential oils may result in additive or synergistic interactions among their constituents, leading to enhanced biological effects compared to those observed for individual oils. This phenomenon, frequently referred to as phytosynergy, holds particular significance in the context of tyrosinase inhibition, where multi-target modulation could improve efficacy and reduce effective dosages. Despite growing interest in plant-derived tyrosinase inhibitors, studies addressing essential oil combinations remain limited. Expanding this research domain may facilitate the development of multifunctional, naturally derived agents for dermatological and cosmetic applications targeting pigmentation disorders. Research into the anti-tyrosinase potential of individual essential oils remains relatively scarce and primarily restricted to in vitro assays [36,37,38]. In our previous investigation [39], we examined essential oil combinations from the rhizomes of *Acorus calamus* (L.) and the cone-berries of *Juniperus communis* (L.). The results demonstrated synergistic tyrosinase inhibition, supporting their potential as natural depigmenting agents. Among the plant species studied, the rhizomes of *Zingiber officinale* (ginger) and the strobiles of *Humulus lupulus* (hop) are noteworthy due to their complex phytochemical profiles and bioactive constituents. Ginger is characterized by pungent phenolic compounds such as gingerols and shogaols, along with volatile terpenes, whereas hops are rich in polyphenols, including xanthohumol and tannins. Although hydroalcoholic and ethanolic extracts of these plants have well-documented bioactivities [40,41,42], the pharmacological effects of their essential oils, especially regarding tyrosinase inhibition, remain insufficiently characterized. Ginger essential oil has been reported to suppress melanin synthesis in B16 murine melanoma cells via downregulation of tyrosinase and other melanogenic genes (e.g., MITF, TYRP1), likely through attenuation of oxidative stress and modulation of intracellular signaling pathways such as MAPK/ERK [40]. These effects are attributed to its lipophilic volatile constituents, including zingiberene, *β*-sesquiphellandrene, and *α*-curcumene. However, these findings are largely limited to cellular models, with a lack of direct enzymatic evidence confirming interaction at the catalytic site of tyrosinase. Conversely, the anti-tyrosinase activity of hop-derived tannins has been more extensively documented. These polyphenols demonstrate mixed-type inhibition of tyrosinase activity, with molecular docking and spectroscopic studies revealing hydrogen bonding to amino acid residues in the enzyme’s active site and copper ion chelation essential for catalytic function [41,42]. Despite the well-established activity of hop polyphenols, no data currently exist on the tyrosinase inhibitory effects of hop essential oil itself. This represents a notable research gap, considering hop essential oil contains sesquiterpenes such as *β*-caryophyllene, myrcene, and humulene, known for their antioxidant and anti-inflammatory properties [43]. The direct impact of these volatiles on tyrosinase activity or melanogenesis remains unexplored. Furthermore, there are no scientific reports examining the combined effects of ginger and hop essential oils on tyrosinase inhibition. Given that essential oils are complex mixtures with potentially multifactorial bioactivities, the possibility of synergistic or additive interactions between these two oils merits further investigation. For instance, the antioxidant capacity and ERK pathway modulation of ginger oil could complement the copper-chelating and enzyme-binding properties of hop volatiles, potentially enhancing overall inhibitory efficacy beyond that of either oil alone. It is also crucial to recognize that essential oils differ markedly from polar plant extracts in physicochemical properties such as polarity, molecular weight distribution, and solubility, which influence bioavailability, cellular uptake, and modes of interaction with enzymes like tyrosinase. Our benchmark study highlights the moderate yet significant tyrosinase-inhibitory effects of ginger (EOZ) and hop (EOH) essential oils and their synergistic combinations. The data clearly demonstrate that EOZ exhibits superior efficacy compared to EOH in tyrosinase inhibition. Additionally, observed synergistic interactions in EOZ:EOH mixtures suggest potential for enhanced bioactivity when these oils are applied in specific ratios.

### 2.3. Lipid Peroxidation Inhibitory Activity of Tested Essential Oils

**Figure 5:**
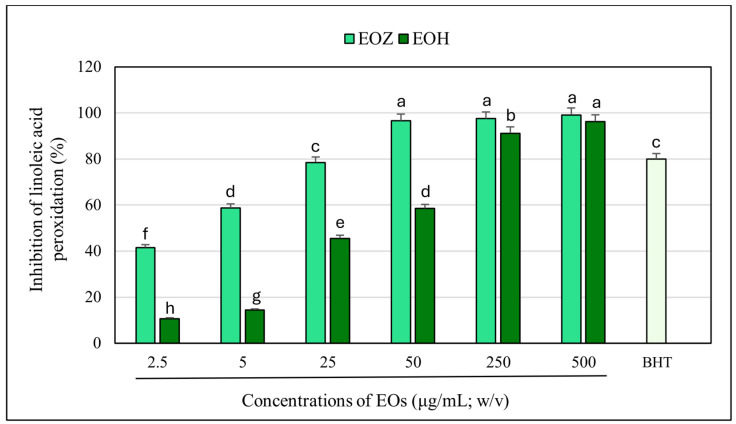
Inhibition of linoleic acid peroxidation by individual essential oils. EOZ and EOH refer to essential oils extracted from *Zingiber officinale* (Rosc.) rhizomes and *Humulus lupulus* (L.) strobiles, respectively. BHT (butylated hydroxytoluene), depicted in light green, at a concentration of 20 μg/mL (*w*/*v*) was included as a positive control. Different letters above the error bars indicate statistically significant differences in mean values (±SD) among the samples, as determined by Tukey’s test (*p* < 0.05).

**Figure 6:**
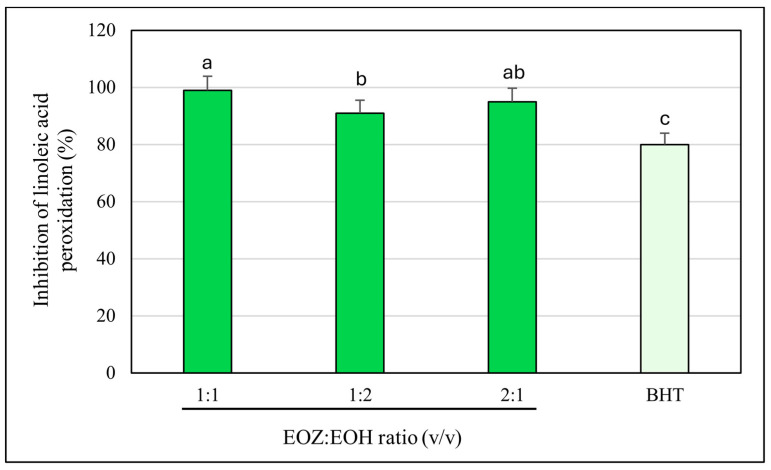
Inhibition of linoleic acid peroxidation by binary mixtures of essential oils isolated from the rhizomes of *Zingiber officinale* Rosc. (EOZ) and the strobiles of *Humulus lupulus* L. (EOH). Three mixtures were formulated using volumetric ratios (*v*/*v*) of 1:1, 1:2, and 2:1 (EOZ:EOH), at a total essential oil concentration of 250 µg/mL (*w*/*v*), corresponding to individual concentrations of 125:125, 83:167, and 167:83 µg/mL, respectively. BHT (butylated hydroxytoluene) at a concentration of 20 μg/mL (*w*/*v*) was included as a positive control. Bars representing the EOZ:EOH mixtures are shown in green, while the bar for the positive control (BHT) is depicted in light green. Different letters above the error bars indicate statistically significant differences between mean values (±SD), as determined by Tukey’s test (*p* < 0.05).

The inhibitory effects of the examined essential oils (EOZ and EOH) on linoleic acid peroxidation were clearly concentration-dependent across the range of 2.5 to 500 µg/mL (Figure 5). Overall, EOZ exhibited significantly greater antioxidant activity compared to EOH. At the highest concentration tested (500 µg/mL), both oils nearly completely inhibited lipid peroxidation, with EOZ achieving slightly superior efficacy. As concentrations decreased, the inhibitory effect of both oils gradually diminished; however, EOZ maintained substantially higher potency than EOH at all lower concentrations. For instance, at 50 µg/mL, EOZ retained over 95% inhibition, whereas EOH’s effect dropped to approximately 59%. At the lowest tested concentration (2.5 µg/mL), EOZ still inhibited lipid peroxidation by more than 40%, in contrast to only about 11% inhibition observed for EOH. The observed dose-dependent inhibition patterns suggest that both essential oils possess antioxidant compounds capable of reducing lipid peroxidation, with EOZ showing a notably higher potency. The reference compound, butylated hydroxytoluene (BHT), used as a positive control at 20 µg/mL (*w*/*v*), exhibited approximately 80% inhibition of linoleic acid peroxidation under the same experimental conditions (Figure 5 and Figure 6). These differences may be attributed to the distinct chemical compositions of the oils, particularly in the content and structure of phenolic constituents known for their radical-scavenging activities. The present study demonstrates that both ginger (EOZ) and hop (EOH) essential oils exhibit notable antioxidant activity, as determined by their ability to inhibit lipid peroxidation in a concentration-dependent manner. The calculated IC_50_ values were 4.2 ± 0.1 µg/mL for EOZ and 39.3 ± 0.5 µg/mL for EOH, confirming the higher antioxidant potency of EOZ. Nevertheless, EOH also showed considerable inhibition across the tested concentrations, confirming its relevance as an effective antioxidant agent. The higher activity of EOZ may be attributed to its rich content of phenolic constituents such as gingerols and shogaols, which are well documented for their radical-scavenging and lipid-stabilizing properties. In turn, the activity observed for EOH is likely driven by compounds such as humulones, lupulones, and prenylated flavonoids, which may contribute to peroxidation inhibition through complementary mechanisms [20,46]. These findings underscore the distinct antioxidant profiles of both oils and support their potential for use as natural inhibitors of lipid peroxidation.

To evaluate the antioxidant activity of EOZ and EOH mixtures, three binary formulations (1:1, 1:2, and 2:1, *v*/*v*) were tested at a total concentration of 250 µg/mL (Figure 6). The 1:1 mixture showed the highest inhibition of linoleic acid peroxidation (99%) with a combination index (CI) of 0.79 ± 0.03, indicating a synergistic interaction (Table S3, Figure 3). This synergy likely results from complementary antioxidant mechanisms of the constituent phytochemicals—such as gingerols and shogaols from ginger oil, and humulones and prenylated flavonoids from hop oil—which may act cooperatively through radical scavenging, regeneration of active antioxidant species, or metal chelation. Mixtures at ratios of 1:2 (91% inhibition) and 2:1 (95%) demonstrated additive antioxidant effects (CI values of 1.01 ± 0.06 and 1.00 ± 0.05, respectively). The lower inhibition observed for the 1:2 ratio may be due to dilution of the more potent ginger oil constituents. The observed synergistic effect at the 1:1 ratio underscores the importance of optimizing the proportion of each essential oil to maximize antioxidant efficacy. The balanced presence of bioactive compounds likely facilitates multiple complementary mechanisms, such as efficient radical neutralization and stabilization of reactive intermediates, which individually might be less effective when the oils are used alone or at non-optimal ratios [20,46]. To further evaluate the dose-sparing effects of these combinations, dose reduction index (DRI) values were calculated. In this assay, DRI values for EOZ ranged from 1.05 to 1.40, whereas EOH displayed substantially higher values (5.6–19.7) (Table S4, Figure 4). These results indicate a slight dose reduction for EOZ and a substantial reduction for EOH when used in combination, highlighting a stronger contribution of EOZ in the mixture. Although this assay provides valuable insight into the antioxidant interactions within the mixtures, it does not account for biological factors such as metabolism, bioavailability, and cellular uptake that influence efficacy in vivo. Nevertheless, the results indicate that a 1:1 (*v*/*v*) ratio of EOZ and EOH provides optimal cooperative antioxidant activity in this assay system, highlighting the need for further investigation in biological models. These results support the potential use of EOZ–EOH mixtures as effective natural antioxidants, with implications for formulations in dermatology, functional foods, and pharmaceuticals aimed at preventing oxidative damage from lipid peroxidation.

The current study emphasizes the importance of assessing the antioxidant potential of essential oils through lipid peroxidation models, particularly those involving linoleic acid. The results of well-characterized oils such as thyme, oregano, and clove underscore the central role of phenolic compounds—such as thymol, carvacrol, and eugenol—in inhibiting lipid oxidation processes [10,47]. These compounds act by donating hydrogen atoms to lipid radicals, thereby terminating the chain reaction of lipid peroxidation and reducing the generation of cytotoxic by-products. In contrast, the antioxidant capacity of ginger and hop essential oils remains underexplored, despite growing evidence that their phytochemical components exhibit free radical-scavenging and anti-inflammatory activities. For instance, ethanolic extracts of ginger have been shown to inhibit peroxidation in sunflower oil systems [3], and essential oil from *Zingiber officinale* demonstrated general antioxidant activity in various in vitro models [48]. However, the limited availability of studies employing linoleic acid as a substrate hampers a full understanding of the oil’s membrane-protective potential. Similarly, despite evidence of antioxidant effects from hop constituents such as xanthohumol [4], the role of hop essential oil in lipid peroxidation remains underexplored. This study thus positions itself at the intersection of phytochemistry and redox biology, aiming to contribute novel data on the peroxidation-inhibitory properties of undercharacterized essential oils. From an applied perspective, uncovering essential oils capable of inhibiting lipid peroxidation may have considerable implications for dermatological formulations aimed at protecting the skin from oxidative damage, as well as for functional food products seeking to enhance oxidative stability. As essential oils are increasingly recognized for their multifunctional bioactivities, the elucidation of their mechanistic antioxidant action at the lipid level becomes a priority. Therefore, elucidating the mechanistic basis of their antioxidant activity at the lipid level is crucial for optimizing their utilization in therapeutic and industrial applications.

### 2.4. ABTS-Based Evaluation of Radical Scavenging Activity of the Investigated Essential Oils

**Figure 7:**
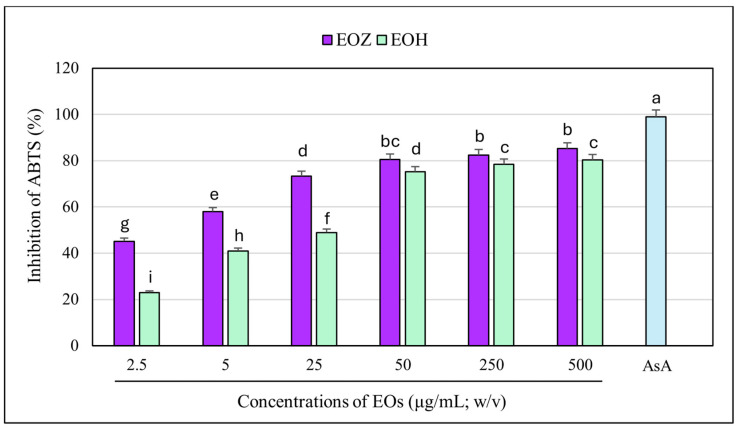
Free radical scavenging activity of the individual essential oils assessed using the ABTS^•+^ assay. EOZ and EOH refer to essential oils extracted from *Zingiber officinale* (Rosc.) rhizomes and *Humulus lupulus* (L.) strobiles, respectively. AsA (ascorbic acid), depicted in the light blue, at a concentration of 150 μg/mL (*w*/*v*) was included as a positive control. Different letters above the error bars represent statistically significant differences in mean antioxidant activity values (± SD) among the samples, as determined by Tukey’s test (*p* < 0.05).

**Figure 8:**
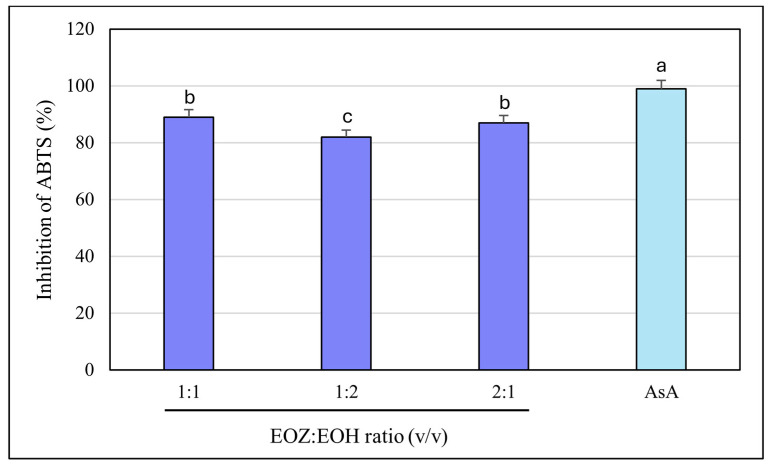
Free radical scavenging activity of binary mixtures of essential oils isolated from the rhizomes of *Zingiber officinale* Rosc. (EOZ) and the strobiles of *Humulus lupulus* L. (EOH), evaluated using the ABTS^•+^ assay. Three mixtures were formulated using volumetric ratios (*v*/*v*) of 1:1, 1:2, and 2:1 (EOZ:EOH), at a total essential oil concentration of 250 µg/mL (*w*/*v*), corresponding to individual concentrations of 125:125, 83:167, and 167:83 µg/mL, respectively. AsA (ascorbic acid) at a concentration of 150 μg/mL (*w*/*v*) was included as a positive control. Bars representing the EOZ:EOH mixtures are shown in dark blue, while the bar for the positive control (AsA) is depicted in light blue. Different letters above the error bars represent statistically significant differences between mean antioxidant activity values (± SD), as determined by Tukey’s test (*p* < 0.05).

The ABTS radical cation decolorization assay (ABTS^•+^) is a widely used and reliable method to assess the antioxidant potential of natural products, including essential oils. It involves the reduction of the blue-green ABTS^•+^ radical by antioxidant compounds, leading to a quantifiable decrease in absorbance at 734 nm. The assay’s high sensitivity and compatibility with both hydrophilic and lipophilic compounds make it particularly suitable for evaluating essential oils [39,49,50]. In this study, both EOZ and EOH demonstrated dose-dependent ABTS^•+^ radical scavenging activity across the tested range (2.5 to 500 µg/mL). As a positive control, ascorbic acid (a commonly used reference antioxidant) was tested at a concentration of 150 μg/mL (*w*/*v*), resulting in nearly 100% inhibition of ABTS^•+^ radical cation (Figure 7 and Figure 8). EOZ displayed superior antiradical scavenging activity across all tested concentrations, with inhibition values ranging from 85.2% to 45.2%, compared to EOH, which exhibited a more modest inhibition effect (80.3–23.0%) (Figure 7). The disparity was particularly pronounced at the lowest concentration (2.5 µg/mL), where EOZ retained nearly half of its scavenging capacity (45.2%) relative to EOH (23.0%). Consistent with these results, IC_50_ values were 2.8 ± 0.04 µg/mL for EOZ and 21.5 ± 0.2 µg/mL for EOH. These findings substantiate the potent antioxidant capacities of both essential oils, with EOZ demonstrating markedly higher efficacy in the ABTS^•+^ radical scavenging assay under the experimental parameters employed. The majority of existing research on the antioxidant properties of *Z. officinale* and *H. lupulus* primarily addresses solvent-based extracts, with comparatively fewer investigations targeting their volatile essential oil fractions. For example, Tarfaoui et al. [51] and Erdoğan [52] reported substantial antioxidant activities of ginger extracts using DPPH, FRAP, and CUPRAC assays; however, these assessments were conducted on polar or hydroalcoholic extracts rather than essential oils. Similarly, studies by Lyu et al. [53] and Di Sotto et al. [54] have focused on hop extracts, revealing cultivar- and extraction-dependent variations in antioxidant capacity, with IC_50_ values ranging from approximately 192 to 451 µg/mL. Notably, few studies have directly evaluated the antioxidant potential of ginger essential oil. One of the rare reports by Höferl et al. [46] documented strong ABTS^•+^ radical scavenging activity for ginger essential oil, corroborating the present study’s observation of significant inhibition by EOZ at higher concentrations. To assess potential synergistic effects, binary mixtures of EOZ and EOH were tested at a total concentration of 250 µg/mL using volume ratios of 1:1, 1:2, and 2:1 (*v*/*v*) (Figure 8). The 1:1 mixture demonstrated the highest ABTS^•+^ radical scavenging activity (89%), followed by the 2:1 (87%) and 1:2 (82%) mixtures. Combination index (CI) values revealed strong synergism for the 1:1 ratio (CI = 0.61 ± 0.03), moderate synergism for the 2:1 ratio (CI = 0.76 ± 0.05), and slight synergism or near-additive effects for the 1:2 ratio (CI = 0.90 ± 0.06) (Table S5, Figure 3). These results indicate that the antioxidant interaction between EOZ and EOH is dependent on their relative proportions, with the most pronounced synergistic effect observed at a 1:1 (*v*/*v*) combination. To further quantify the practical implications of these synergistic effects, dose reduction index (DRI) values were calculated. DRI values for EOZ varied between 1.4 and 1.9, indicating a mild dose-sparing effect, while those for EOH ranged from about 5.4 to 21.5, evidencing significant dose-sparing effects for EOH when combined with EOZ (Table S6, Figure 4). Collectively, these data indicate that both EOZ and EOH possess significant antioxidant potential, with EOZ exhibiting superior radical scavenging activity in the ABTS^•+^ assay. In addition, the binary combinations of these essential oils—especially at an equal ratio—enhance antioxidant activity through synergistic interactions. This underscores the necessity for further research to elucidate the mechanistic basis, efficacy, and practical applications of essential oil-based antioxidant formulations [55,56].

The evaluation of antiradical activity in essential oils is crucial due to their potential role in mitigating oxidative stress, a fundamental pathological mechanism implicated in the etiology of numerous chronic diseases, including neurodegenerative disorders, cardiovascular diseases, and cancer [6,8]. Oxidative stress arises from an imbalance between reactive oxygen species (ROS) generation and endogenous antioxidant defenses, leading to cellular damage and inflammation [5]. In dermatology, oxidative stress is recognized as a key pathophysiological factor contributing to skin disorders such as photoaging, acne, atopic dermatitis, and psoriasis [57,58]. Essential oils are complex mixtures of volatile compounds—including terpenes, phenylpropanoids, and alcohols—that exhibit antioxidant activities through diverse mechanisms such as hydrogen atom donation, radical scavenging, and metal ion chelation. These mechanisms act synergistically to neutralize free radicals and attenuate oxidative damage [9,10]. The presence of phenolic constituents in essential oils is particularly significant due to their conjugated double bonds and hydroxyl groups, which facilitate electron transfer and potentiate antioxidant efficacy [47]. In dermatological contexts, the antioxidant properties of essential oils contribute to cellular protection against oxidative damage induced by environmental stressors such as ultraviolet (UV) radiation and pollution. For example, rosemary (*Rosmarinus officinalis*) essential oil has been shown to reduce UV-induced skin damage by lowering ROS levels and modulating inflammatory pathways [59]. Similarly, chamomile (*Matricaria chamomilla*) and lavender (*Lavandula angustifolia*) essential oils demonstrate both anti-inflammatory and antioxidant activities, which may alleviate symptoms of inflammatory skin diseases such as eczema and psoriasis [60,61]. Moreover, essential oils with antioxidant properties may enhance epidermal barrier integrity, stimulate collagen synthesis, and accelerate tissue repair, thereby supporting dermal homeostasis and facilitating the treatment of various skin conditions. For instance, rosehip (*Rosa canina*) seed oil—rich in vitamin C and essential fatty acids—has been reported to reduce hyperpigmentation and improve skin elasticity, contributing to scar healing and anti-aging effects [62]. Mechanistic insights into EO antioxidant effects will enable the development of targeted interventions for the prevention and management of oxidative stress-related skin disorders, ultimately enhancing skin health and overall physiological function.

## 3. Materials and Methods

### 3.1. Plant Material and Essential Oil Extraction

The essential oils (EOs) analyzed in this study were obtained from the rhizomes of *Zingiber officinale* Rosc. (ginger) and the strobiles of *Humulus lupulus* L. (hop). Both plant materials were acquired from the same commercial supplier (NANGA, Blękwit, Poland) and were accompanied by certificates of analysis. According to the supplier’s documentation, the raw materials complied with established quality standards regarding moisture and ash levels, the presence of contaminants (including ochratoxin A and heavy metals), microbiological purity, and organoleptic characteristics. Specifically, the moisture content was below the permissible limit of 10%, and the total ash content did not exceed 20%. Heavy metal concentrations were within acceptable limits, with lead (Pb) below 3 ppm and cadmium (Cd) below 1 ppm. Ochratoxin A was present at a level not exceeding 10 µg/kg. Microbiological testing confirmed the absence of *Salmonella* spp. The total aerobic microbial count (TAMC) remained below 10^7^ CFU/g, while yeast and mold counts were below the commonly accepted threshold of 10^6^ CFU/g, indicating appropriate storage and handling conditions. The ginger rhizomes (batch no. 18324/2336/118747/1) originated from Nigeria, whereas the hop strobiles (batch no. PZ/2024-052) were sourced domestically from Poland (NANGA, Blękwit, Poland). All plant materials were harvested during the 2024 growing season. Prior to essential oil extraction, both plant materials were dried to a final moisture level of less than 10%. Hop strobiles were dried at room temperature (approximately 20–22 °C) in a well-ventilated room with continuous air circulation. Ginger rhizomes were sliced into thin pieces and arranged in a single layer on trays, then dried in a cabinet dryer at 40 °C with forced air circulation. After drying, the plant materials were ground into a fine powder (20–40 mesh) using a laboratory mill.

For essential oil isolation, 40 g portions of each powdered sample were subjected to hydrodistillation for 3 h using a Clevenger-type apparatus (catalogue no. ROTH XX23.1; Carl ROTH GmbH + Co. KG, Karlsruhe, Germany) at approximately 100 °C under atmospheric pressure. The distilled essential oils were subsequently dried over anhydrous sodium sulfate, filtered, and stored in amber glass vials at 4 °C, protected from light and air, until further use [39].

### 3.2. Preparation of Essential Oil Samples

To assess the anti-tyrosinase and antioxidant activities of the examined essential oils, six concentrations (2.5, 5, 25, 50, 250, and 500 µg/mL; *w*/*v*) were prepared by dissolving the oils in ethanol. Three binary mixtures of ginger (EOZ) and hop (EOH) essential oils were formulated using volume ratios (*v*/*v*) of 1:1, 1:2, and 2:1, at a total concentration of 250 µg/mL (*w*/*v*). These ratios corresponded to individual oil concentrations of 125:125, 83:167, and 167:83 µg/mL (EOZ:EOH), respectively.

All samples were freshly prepared before each assay and vortexed thoroughly to ensure homogeneity. In preliminary trials, higher concentrations of the individual essential oils were also prepared; however, visible emulsification occurred in the ethanolic solvent system, particularly after mixing the two oils. This effect was more pronounced in the binary formulations than in the single-oil solutions, leading to the formation of turbid emulsions that were unsuitable for spectrophotometric analysis. Conversely, lower concentrations (2.5–500 µg/mL) of single essential oils yielded optically clear and stable solutions. For the combinatorial analysis, a final concentration of 250 µg/mL was selected, as it represented the highest level at which all tested mixtures remained homogeneous and analytically acceptable.

### 3.3. Gas Chromatography-Mass Spectrometry (GC-MS) Characterization of Essential Oil Components

The chemical constituents of the tested essential oils (EOZ and EOH) were identified and quantified by gas chromatography–mass spectrometry (GC-MS) following the procedure previously described by Sytykiewicz et al. [39]. Briefly, analyses were performed using a TRACE GC-ULTRA system coupled with a Polaris Q mass spectrometer (Thermo Fisher Scientific, Waltham, MA, USA), equipped with an Equity-5 capillary GC column (Supelco) and utilizing electron impact ionization at 70 eV. Compound identification was based on comparisons of retention indices and mass spectra with the literature data and the NIST Mass Spectral Library (National Institute of Standards and Technology, Gaithersburg, MD, USA) [63,64].

### 3.4. Evaluation of the Anti-Tyrosinase Activity of Essential Oils

The tyrosinase inhibitory activity of the essential oils (EOs) was determined using the Tyrosinase Inhibitor Screening Assay Kit (catalogue no. ab204715, Abcam, Cambridge, UK), with minor modifications based on the procedure described by Sytykiewicz et al. [39]. Each EO sample was prepared at the designated concentration by diluting it in a premix containing 2 µL of 96% ethanol, 1 µL of DMSO (dimethyl sulfoxide), and Tyrosinase Assay Buffer up to a total volume of 20 µL. The reaction mixture was composed of 20 µL of the EO sample, 50 µL of Tyrosinase Enzyme Solution, and 30 µL of Tyrosine/Tyrosinase Substrate Solution, yielding a total volume of 100 µL. This setup resulted in final solvent concentrations of approximately 1.92% (*v*/*v*) ethanol and 1% (*v*/*v*) DMSO. An enzyme control (EC), lacking essential oil, was included in the assay. Kojic acid was dissolved in double-distilled water (ddH_2_O) to a final concentration of 710 μg/mL (*w*/*v*) and used as the inhibitor control (IC). The solvent control (SC) consisted of 2 µL of 96% ethanol, 1 µL of DMSO, and 17 µL of Tyrosinase Assay Buffer (total 20 µL). After adding the SC to the reaction mixture, the final solvent percentages were consistent with those in the samples. All mixtures were incubated at 25 °C for 90 min, followed by absorbance measurement at 510 nm using a microplate UV–Vis spectrophotometer (Epoch, BioTek, Winooski, VT, USA). The enzyme inhibition percentage was calculated as follows:

% Relative Inhibition = [(Slope of EC − Slope of S)/Slope of EC] × 100

where

EC—the enzyme control;

S—the sample.

### 3.5. Assessment of Linoleic Acid Peroxidation Inhibition by Examined Essential Oils

The ability of essential oils to inhibit linoleic acid peroxidation was evaluated using the method described by Kuo et al. [65], with slight modifications. Briefly, 10 µL of either ginger or hop essential oil was added to 370 µL of 0.05 M phosphate buffer (pH 7.0) containing 0.05% Tween 20 and 4 mM linoleic acid. Ethanol served as the negative control. The mixtures were pre-incubated at 37 °C for 3 min. Lipid peroxidation was initiated by the addition of 20 µL of 0.035% hemoglobin solution prepared in deionized water. Subsequently, the samples were incubated at 37 °C in a shaking incubator at 120 rpm for 10 min. The reaction was terminated by the addition of 5 mL of 0.6% HCl in ethanol.

The extent of lipid peroxidation was determined using the ferric thiocyanate colorimetric assay. Specifically, 0.1 mL of 20 mM FeCl_2_ and 0.1 mL of 30% ammonium thiocyanate were added to the reaction mixtures, and absorbance was measured at 480 nm. A solution of BHT (butylated hydroxytoluene) at a concentration of 20 μg/mL in ethanol (*w*/*v*) was prepared freshly prior to each experiment and used as the positive control.

The antioxidant activity of the EOs was calculated using the following equation:

Inhibition effect (%) = [1 − (A_S_ − A_0_)/(A_C_ − A_0_)] × 100

where

A_0_—absorbance of the sample (EO or BHT) without hemoglobin;

A_C_—absorbance of the negative control reaction (containing ethanol instead of the tested sample);

A_S_—absorbance of the reaction mixture (containing the EO or BHT).

The IC_50_ value—defined as the concentration of essential oil (μg/mL) required to inhibit 50% of linoleic acid peroxidation—was subsequently determined.

### 3.6. Antioxidant Capacity of Essential Oils Using the ABTS^•+^ Radical Scavenging Assay

The antioxidant activity of the analyzed essential oils against the ABTS^•+^ radical cation (2,2′-azinobis(3-ethylbenzothiazoline-6-sulfonic acid)) was assessed according to a modified method described by Re et al. [49]. The ABTS^•+^ was generated by combining an aqueous ABTS solution (7 mM) with potassium persulfate (2.45 mM) in a 1:0.5 volume ratio, followed by incubation in the dark at room temperature for 16 h. Prior to testing, the ABTS^•+^ stock solution was diluted with 96% ethanol to reach an absorbance of approximately 0.7 at 734 nm. A freshly prepared solution of ascorbic acid (AsA) in double-distilled water at a concentration of 150 μg/mL (*w*/*v*) was used as a positive control. For the assay, 350 μL of ABTS^•+^ working solution was combined with 50 μL of the EO sample containing 0.5% Tween 20 (*v*/*v*) to ensure clarity and proper mixing. After vortexing for 15 s, the mixture was kept in the dark at room temperature for 30 min to allow the reaction to proceed. Absorbance readings of the control ABTS^•+^ solution (A_0_) and the EO-treated or positive control samples (A_S_) were recorded at 734 nm using a UV–Vis microplate spectrophotometer (Epoch, BioTek, Winooski, VT, USA). The percentage of radical scavenging activity was calculated as follows:

ABTS^•+^ inhibition (%) = [(A_0_ − A_S_)/A_0_] × 100

where

A_0_—absorbance of the control ABTS^•+^ solution;

A_S_—absorbance of the ABTS^•+^ solution treated with EO sample or positive control (ascorbic acid).

Subsequently, the IC_50_ value, defined as the concentration of EO (μg/mL) needed to scavenge 50% of ABTS^•+^ cation radicals, was assessed.

### 3.7. Evaluation of Interaction Effects Between Ginger and Hop Essential Oils Using Combination Index (CI) and Dose Reduction Index (DRI) Analyses

The interaction effects between essential oils extracted from *Zingiber officinale* Rosc. rhizomes (EOZ) and *Humulus lupulus* L. strobiles (EOH) were evaluated using the combination index (CI) and dose reduction index (DRI) values, calculated in accordance with the Chou–Talalay method [33,44,45]. CI and DRI values were visualized using heatmaps to illustrate the interaction type and the extent of dose reduction in three cell-free in vitro assays evaluating anti-tyrosinase activity, linoleic acid peroxidation inhibition, and ABTS^•+^ scavenging efficacy. The combination index (CI; mean ± SD) was calculated using the following equation:

CI = (D_1_/Dx_1_) + (D_2_/Dx_2_)

where

D_1_ and D_2_ denote the concentrations of EOZ and EOH, respectively, in the mixture required to achieve a defined inhibitory effect;

Dx_1_ and Dx_2_ represent the concentrations of EOZ and EOH, respectively, needed to achieve the same effect when used individually.

CI values were interpreted as follows: CI < 1 indicates synergy, CI ≈ 1 indicates additivity, and CI > 1 denotes antagonism. The dose reduction index (DRI; mean ± SD) was calculated individually for EOZ and EOH using the following equation:

DRI = Dx/D

where

Dx—the dose of essential oil required to achieve a given inhibitory effect when used individually;

D—the dose of essential oil in combination needed to achieve the same level of inhibition.

DRI > 1 indicates a beneficial dose reduction achieved by combining EOZ and EOH, meaning that a lower concentration of the individual oil is required in the combination to achieve the same effect as when used alone. The magnitude of reduction is expressed as *n*-fold (e.g., DRI = 2 corresponds to a two-fold dose reduction). DRI ≈ 1 reflects no dose reduction, while DRI < 1 suggests that a higher dose is needed in combination, implying a less effective interaction.

All data analyses and visualizations were performed using Python version 3.11.0 (Python Software Foundation, Wilmington, DE, USA) [66]. Data processing and plotting employed the following libraries: pandas (version 2.3.1) for data handling, seaborn (version 0.13.2) for statistical data visualization, and matplotlib (version 3.10.3) for generating plots. Heatmaps were used to illustrate the CI and DRI values for EOZ and EOH at different mixture ratios (1:1, 1:2, and 2:1; *v*/*v*), providing a clear visual overview of the interaction effects.

### 3.8. Statistical Analysis

Results are expressed as mean values ± standard deviation (SD). The study included three independent experimental series, each with two technical replicates. Differences in the measured parameters (e.g., anti-tyrosinase activity, inhibition of linoleic acid peroxidation, ABTS^•+^ radical scavenging) between the two essential oils and their mixtures were evaluated by an analysis of variance (ANOVA) followed by Tukey’s post hoc test. A *p*-value below 0.05 was considered statistically significant. All analyses were conducted using Statistica version 13.3 software (Statsoft, Kraków, Poland).

## 4. Conclusions

The comprehensive GC–MS analysis revealed distinct chemotypic differences between EOZ and EOH, with EOZ characterized by a predominance of sesquiterpenes such as *α*-zingiberene, and EOH dominated by oxygenated sesquiterpenes including *β*-caryophyllene diols. Both essential oils demonstrated dose-dependent inhibition of tyrosinase activity, lipid peroxidation, and ABTS^•+^ radical scavenging, with EOZ consistently exhibiting stronger bioactivity across all three non-cellular in vitro assays. Importantly, binary mixtures of EOZ and EOH resulted in notable synergistic interactions, as evidenced by combination index (CI) values < 1 for most formulations. These interactions were particularly pronounced in anti-tyrosinase and ABTS^•+^ assays. In addition, dose reduction index (DRI) analysis confirmed that combining EOZ and EOH lowered the concentrations required for each oil to achieve equivalent effects. The most substantial reductions were observed for EOH, indicating its enhanced efficacy in the presence of EOZ. This multi-fold dose reduction suggests complementary mechanisms of action between the oils and highlights the advantage of their combination over individual application. Collectively, these findings underscore the potential of EOZ and EOH as promising candidates for the development of multifunctional antioxidant and enzyme-inhibitory agents. The synergistic interactions demonstrated not only improved the combination index values but also led to significant dose reductions, thereby enhancing the overall functional activity. Further studies in cellular and in vivo models are warranted to validate these interactions and assess bioavailability, safety, and effectiveness in more complex biological systems.

## Figures and Tables

**Table 1:** GC-MS profiling and identification of chemical constituents in the essential oil of *Zingiber officinale* (Rosc.) rhizomes.

Chemical Compounds	RT	RI	Peak Area (%)
2-Heptanol	9.84	901	0.1
Tricyclene	10.55	919	0.1
*α*-Thujene	10.79	926	<0.1
*α*-Pinene	11.00	931	2.8
Camphene	11.56	946	6.8
Sabinene	12.55	971	0.1
*β*-Pinene	12.64	974	0.4
Sulcatone	13.13	986	0.2
*β*-Myrcene	13.30	991	1.4
*α*-Phellandrene	13.75	1003	0.3
Δ3-Carene	13.98	1009	0.1
*α*-Terpinene	14.23	1015	<0.1
*p*-Cymene	14.53	1023	0.2
*β*-Phellandrene	14.69	1027	5.7
1,8-Cineole	14.77	1029	4.8
*γ*-Terpinene	15.88	1058	<0.1
Terpinolene	17.05	1088	0.3
2-Nonanone	17.16	1091	<0.1
Pinene oxide	17.35	1096	0.1
Rosefuran	17.36	1096	0.2
Linalool	17.46	1099	0.7
(*Z*)-*p*-Menth-2-en-1-ol	18.24	1120	<0.1
cis-*p*-Mentha-2,8-dien-1-ol	18.93	1139	<0.1
Camphor	19.09	1143	0.1
*trans*-Verbenol	19.13	1144	<0.1
(*E*)-*p*-Menth-2-en-1-ol	19.24	1147	<0.1
*β*-Citronellal	19.44	1153	0.3
Isoborneol	19.57	1156	<0.1
Borneol	19.90	1165	1.0
Rosefuran epoxide	20.26	1175	0.1
Terpinen-4-ol	20.33	1177	0.1
Isogeranial	20.53	1182	0.1
*p*-Cymen-8-ol	20.65	1185	0.1
*α*-Terpineol	20.82	1190	0.9
Myrtenal	21.01	1195	0.1
Methyl chavicol	21.10	1198	0.1
*β*-Citronellol	22.17	1228	0.6
Nerol	22.36	1233	<0.1
Neral	22.65	1242	7.2
Geraniol	23.09	1254	0.4
(*E*)-2-Decenal	23.34	1261	0.1
Geranial	23.71	1272	12.7
Bornyl acetate	24.22	1286	0.1
2-Undecanone	24.45	1293	0.1
*δ*-Elemene	25.99	1339	<0.1
Citronellyl acetate	26.46	1353	<0.1
*α*-Ylangene	26.93	1367	0.2
*α*-Copaene	27.27	1377	0.5
Geranyl acetate	27.45	1383	<0.1
*β*-Cubebene	27.75	1392	0.1
*β*-Elemene	27.80	1393	0.5
(*E*)-*β*-Caryophyllene	28.69	1421	0.1
*β*-Copaene	28.74	1423	0.1
*γ*-Elemene	29.12	1435	0.2
(*E*)-*α*-Bergamotene	29.18	1437	<0.1
*allo*-Aromadendrene	30.01	1460	0.3
*γ*-Muurolene	30.46	1478	0.5
*α*-Curcumene	30.63	1483	7.8
*β*-Selinene	30.81	1489	0.1
*α*-Zingiberene	31.06	1497	11.4
*β*-Bisabolene	31.42	1509	8.9
*γ*-Cadinene	31.59	1515	0.5
*trans*-Calamenene	31.90	1525	0.3
*β*-Sesquiphellandrene	31.92	1526	5.4
Elemol	32.68	1552	0.5
Zingiberenol (isomer 1)	34.55	1616	0.7
Zingiberenol (isomer 2)	35.03	1633	1.1
*β*-Eudesmol (isomer 1)	35.64	1654	0.5
*β*-Eudesmol (isomer 2)	35.74	1658	0.4
(*Z*)-Nuciferal	37.51	1721	0.2
(*E*)-Nuciferal	37.73	1729	0.1
**Total**	-	-	**89.3**

GC-MS—gas chromatography–mass spectrometry; RT—retention time (min); RI—retention index.

**Table 2:** GC-MS profiling and identification of chemical constituents in the essential oil of *Humulus lupulus* (L.) strobiles.

Chemical Compounds	RT	RI	Peak Area (%)
Methyl isobutyl ketone	4.66	670	1.8
3-Methyl-2-pentanone	4.99	710	0.5
*α*-Pinene	11.00	931	0.5
2-Butyl-1,3,3-trimethylcyclohexene	13.74	1002	0.5
Limonene	14.69	1027	0.2
*cis*-Linalool oxide	16.40	1071	0.4
*trans*-Linalool oxide	17.01	1087	0.3
Linalool	17.46	1099	0.5
3-Carene-10-al	17.54	1101	0.4
*α*-Fenchol	17.94	1112	0.2
Borneol	19.92	1166	0.5
*α*-Terpineol	20.82	1190	0.2
*trans*-Anethole	24.21	1286	7.5
2-Undecanone	24.45	1293	2.1
*α*-Ylangene	27.12	1373	0.7
*α*-Copaene	27.27	1377	1.7
(*E*)-*α*-Bergamotene	29.18	1437	1.9
*α*-Humulene	29.77	1456	0.8
*γ*-Muurolene	30.48	1479	3.5
Germacrene D	30.63	1483	0.9
*β*-Selinene	30.81	1489	1.3
*α*-Selinene	31.07	1497	1.2
*γ*-Cadinene	31.64	1517	4.8
*trans*-Calamenene	31.90	1525	4.3
*α*-Cadinene	32.33	1540	0.6
*α*-Calacorene	32.51	1546	1.0
*β*-Elemenone	34.02	1597	0.8
*trans*-Caryophyllene oxide	34.18	1602	2.2
1,2-Humulenepoxide	34.49	1613	2.3
1,10-di-*epi*-Cubenol	34.63	1618	0.6
*epi*-*α*-Cadinol	35.36	1644	1.8
*α*-Cadinol	35.76	1658	1.1
Cadalene	36.32	1678	2.1
1-*epi*-Cubenol	37.85	1734	12.2
*β*-Caryophyllene-9,10-diol (isomer 1)	40.12	1819	1.9
*β*-Caryophyllene-9,10-diol (isomer 2)	40.30	1826	12.7
**Total**	-	-	**71.6**

GC-MS—gas chromatography–mass spectrometry; RT—retention time (min); RI—retention index.

## Data Availability

The data presented in the study are available in the article.

## Supplementary Materials

The following supporting information can be downloaded at https://www.mdpi.com/article/10.3390/molecules30153294/s1, Figure S1: GC-MS chromatogram of the essential oil isolated from *Zingiber officinale* (Rosc.) rhizomes; Figure S2: GC-MS chromatogram of the essential oil isolated from *Humulus lupulus* (L.) strobiles; Table S1. Combination index (CI) values representing the interaction effects of EOZ and EOH mixtures on tyrosinase activity inhibition; Table S2. Dose reduction index (DRI) values for binary mixtures of EOZ and EOH essential oils in the anti-tyrosinase activity assay evaluated in a non-cellular in vitro system; Table S3. Combination index (CI) values representing the interaction effects of EOZ and EOH mixtures on linoleic acid peroxidation inhibition; Table S4. Dose reduction index (DRI) values for binary mixtures of EOZ and EOH essential oils in the linoleic acid peroxidation inhibition assay evaluated in a non-cellular in vitro system; Table S5. Combination index (CI) values representing the interaction effects of EOZ and EOH mixtures on ABTS^•+^ radical scavenging activity; Table S6. Dose reduction index (DRI) values for binary mixtures of EOZ and EOH essential oils in ABTS^•+^ radical scavenging assay evaluated in a non-cellular in vitro system.
