# Supplementary material for: New Insights into the Synergistic Bioactivities of Zingiber officinale (Rosc.) and Humulus lupulus (L.) Essential Oils: Targeting Tyrosinase Inhibition and Antioxidant Mechanisms
Source: Molecules. 2025 Aug 6;30(15):3294. doi: 10.3390/molecules30153294 (PMC12348251; doi:10.3390/molecules30153294)
Supplement: Supplementary file 1 [file molecules-30-03294-s001.zip › Table S1.pdf]

Table S1. Combination Index (CI) values representing the interaction effects of EOZ and EOH mixtures on tyrosinase activity inhibition.

Formulation Ratio (EOZ:EOH, v/v)	D_1 (EOZ, μg/mL)	D_2 (EOH, μg/mL)	Dx_1 (IC_{20} of EOZ, μg/mL)	Dx_2 (IC_{20} of EOH, μg/mL)	D_1/Dx_1 (EOZ)	D_2/Dx_2 (EOH)	CI	Interaction Effect
1:1	1.75±0.05	1.75±0.04	3.00±0.12	41.00±1.80	0.58±0.02	0.04±0.002	**0.62±0.02 b**	Synergism
1:2	2.00±0.10	4.00±0.15	3.00±0.12	41.00±1.80	0.67±0.03	0.10±0.002	**0.77±0.05 a**	Synergism
2:1	1.50±0.07	0.75±0.03	3.00±0.12	41.00±1.80	0.50±0.02	0.02±0.001	**0.52±0.01 c**	Synergism

EOZ and EOH represent essential oils isolated from *Zingiber officinale* (Rosc.) rhizomes and *Humulus lupulus* (L.) strobiles, respectively. Mixtures were prepared at volume ratios of EOZ to EOH (v/v): 1:1, 1:2, and 2:1. Due to the lack of 50% inhibition of tyrosinase activity by the tested essential oils, the half-maximal inhibitory concentration (IC_{50}) could not be determined. Instead, the IC_{20} (concentration required for 20% inhibition) for each essential oil was calculated to compare their inhibitory potential against tyrosinase. D_1 and D_2 denote the concentrations of EOZ and EOH, respectively, in the mixture required to cause 20% inhibition of the tyrosinase activity. Dx_1 and Dx_2 represent the concentrations of EOZ and EOH, respectively, needed to achieve the 20% level of inhibition (IC_{20}), when used individually. CI values (mean ± SD) were calculated based on the median-effect principle using the Chou–Talalay method [33]. Interpretation: CI < 1 indicates synergism, CI ≈ 1 additive effect, and CI > 1 antagonism. Different letters represent statistically significant differences between CI values, as determined by Tukey's test ($p < 0.05$).
