# Supplementary material for: New Insights into the Synergistic Bioactivities of Zingiber officinale (Rosc.) and Humulus lupulus (L.) Essential Oils: Targeting Tyrosinase Inhibition and Antioxidant Mechanisms
Source: Molecules. 2025 Aug 6;30(15):3294. doi: 10.3390/molecules30153294 (PMC12348251; doi:10.3390/molecules30153294)
Supplement: Supplementary file 1 [file molecules-30-03294-s001.zip › Table S2.pdf]

Table S2. Dose Reduction Index (DRI) values for binary mixtures of EOZ and EOH essential oils in the anti-tyrosinase activity assay evaluated in a non-cellular *in vitro* system.

Formulation Ratio (EOZ:EOH, v/v)	D_1 (EOZ, µg/mL)	D_2 (EOH, µg/mL)	Dx_1 (IC_{20} of EOZ, µg/mL)	Dx_2 (IC_{20} of EOH, µg/mL)	DRI (Dx_1/D_1) (EOZ)	DRI (Dx_2/D_2) (EOH)
1:1	1.75±0.05	1.75±0.04	3.00±0.12	41.00±1.80	**1.71±0.04 a**	**23.43±0.32 b**
1:2	2.00±0.10	4.00±0.15	3.00±0.12	41.00±1.80	**1.50±0.06 ab**	**10.25±0.48 c**
2:1	1.50±0.07	0.75±0.03	3.00±0.12	41.00±1.80	**2.00±0.07 a**	**54.67±2.05 a**

EOZ and EOH represent essential oils isolated from *Zingiber officinale* (Rosc.) rhizomes and *Humulus lupulus* (L.) strobiles, respectively. Mixtures were prepared at volume ratios of EOZ to EOH (v/v): 1:1, 1:2, and 2:1. Due to the lack of 50% inhibition of tyrosinase activity by the tested essential oils, the half-maximal inhibitory concentration (IC_{50}) could not be determined. Instead, the IC_{20} (concentration required for 20% inhibition) for each essential oil was calculated to compare their inhibitory potential against tyrosinase. D_1 and D_2 denote the concentrations of EOZ and EOH, respectively, in the mixture, required to cause 20% inhibition of the tyrosinase activity. Dx_1 and Dx_2 represent the concentrations of EOZ and EOH, respectively, needed to achieve the 20% level of inhibition (IC_{20}), when used individually. DRI values were calculated using the Chou–Talalay method [33,44,45], according to the following formula: DRI = Dx/D. Interpretation: DRI > 1 indicates a dose reduction achieved by combining EOZ and EOH, meaning that a lower concentration of the individual oil is required in the mixture to elicit the same effect as when used alone. The magnitude of reduction is expressed as *n*-fold (e.g., DRI = 2 corresponds to a two-fold dose reduction). DRI ≈ 1 reflects no dose reduction (i.e., additive effect), while DRI < 1 suggests an antagonistic interaction, where a higher concentration is needed in combination than alone. Different letters represent statistically significant differences between DRI values, as determined by Tukey's test ($p < 0.05$).
