# Supplementary material for: New Insights into the Synergistic Bioactivities of Zingiber officinale (Rosc.) and Humulus lupulus (L.) Essential Oils: Targeting Tyrosinase Inhibition and Antioxidant Mechanisms
Source: Molecules. 2025 Aug 6;30(15):3294. doi: 10.3390/molecules30153294 (PMC12348251; doi:10.3390/molecules30153294)
Supplement: Supplementary file 1 [file molecules-30-03294-s001.zip › Table S3.pdf]

**Table S3.** Combination Index (CI) values representing the interaction effects of EOZ and EOH mixtures on linoleic acid peroxidation inhibition.

| Formulation Ratio (EOZ:EOH, v/v) | $D_1$ (EOZ, µg/mL) | $D_2$ (EOH, µg/mL) | $Dx_1$ ($IC_{50}$ of EOZ, µg/mL) | $Dx_2$ ($IC_{50}$ of EOH, µg/mL) | $D_1/Dx_1$ (EOZ) | $D_2/Dx_2$ (EOH) | CI | Interaction Effect |
|---|---|---|---|---|---|---|---|---|
| 1:1 | 3.00±0.15 | 3.00±0.15 | 4.20±0.10 | 39.30±0.50 | 0.71±0.02 | 0.08±0.006 | **0.79±0.03 b** | Synergism |
| 1:2 | 3.50±0.18 | 7.00±0.24 | 4.20±0.10 | 39.30±0.50 | 0.83±0.04 | 0.18±0.012 | **1.01±0.06 a** | Additive |
| 2:1 | 4.00±0.22 | 2.00±0.05 | 4.20±0.10 | 39.30±0.50 | 0.95±0.05 | 0.05±0.003 | **1.00±0.05 a** | Additive |

EOZ and EOH represent essential oils isolated from *Zingiber officinale* (Rosc.) rhizomes and *Humulus lupulus* (L.) strobiles, respectively. Mixtures were prepared at volume ratios of EOZ to EOH (v/v): 1:1, 1:2, and 2:1. $D_1$ and $D_2$ denote the concentrations of EOZ and EOH, respectively, in the mixture, required to cause 50% inhibition of linoleic acid peroxidation. $Dx_1$ and $Dx_2$ represent the concentrations of EOZ and EOH, respectively, needed to achieve the 50% level of inhibition ($IC_{50}$), when used individually. CI values (mean ± SD) were calculated based on the median-effect principle using the Chou–Talalay method [33]. Interpretation: CI < 1 indicates synergism, CI ≈ 1 additive effect, and CI > 1 antagonism. Different letters represent statistically significant differences between CI values, according to Tukey's test ($p < 0.05$).
