# Supplementary material for: New Insights into the Synergistic Bioactivities of Zingiber officinale (Rosc.) and Humulus lupulus (L.) Essential Oils: Targeting Tyrosinase Inhibition and Antioxidant Mechanisms
Source: Molecules. 2025 Aug 6;30(15):3294. doi: 10.3390/molecules30153294 (PMC12348251; doi:10.3390/molecules30153294)
Supplement: Supplementary file 1 [file molecules-30-03294-s001.zip › Table S4.pdf]

**Table S4.** Dose Reduction Index (DRI) values for binary mixtures of EOZ and EOH essential oils in the linoleic acid peroxidation inhibition assay evaluated in a non-cellular *in vitro* system.

| Formulation Ratio (EOZ:EOH, v/v) | $D_1$ (EOZ, µg/mL) | $D_2$ (EOH, µg/mL) | $Dx_1$ ($IC_{50}$ of EOZ, µg/mL) | $Dx_2$ ($IC_{50}$ of EOH, µg/mL) | DRI ($Dx_1/D_1$) (EOZ) | DRI ($Dx_2/D_2$) (EOH) |
|---|---|---|---|---|---|---|
| 1:1 | 3.00±0.15 | 3.00±0.15 | 4.20±0.10 | 39.30±0.50 | **1.40±0.03 a** | **13.10±0.55 b** |
| 1:2 | 3.50±0.18 | 7.00±0.24 | 4.20±0.10 | 39.30±0.50 | **1.20±0.02 a** | **5.61±0.38 c** |
| 2:1 | 4.00±0.22 | 2.00±0.05 | 4.20±0.10 | 39.30±0.50 | **1.05±0.02 a** | **19.65±0.72 a** |

EOZ and EOH represent essential oils isolated from *Zingiber officinale* (Rosc.) rhizomes and *Humulus lupulus* (L.) strobiles, respectively. Mixtures were prepared at volume ratios of EOZ to EOH (v/v): 1:1, 1:2, and 2:1. $D_1$ and $D_2$ denote the concentrations of EOZ and EOH, respectively, in the mixture required to cause 50% inhibition of linoleic acid peroxidation. $Dx_1$ and $Dx_2$ represent the concentrations of EOZ and EOH, respectively, needed to achieve the 50% inhibition ($IC_{50}$), when used individually. DRI values were calculated using the Chou–Talalay method [33,44,45], according to the following formula: DRI = Dx/D. Interpretation: DRI > 1 indicates a dose reduction achieved by combining EOZ and EOH, meaning that a lower concentration of the individual oil is required in the mixture to elicit the same effect as when used alone. The magnitude of reduction is expressed as *n*-fold (e.g., DRI = 2 corresponds to a two-fold dose reduction). DRI ≈ 1 reflects no dose reduction (i.e., additive effect), while DRI < 1 suggests an antagonistic interaction, where a higher concentration is needed in combination than alone. Different letters represent statistically significant differences between DRI values, as determined by Tukey's test ($p < 0.05$).
