# Supplementary material for: New Insights into the Synergistic Bioactivities of Zingiber officinale (Rosc.) and Humulus lupulus (L.) Essential Oils: Targeting Tyrosinase Inhibition and Antioxidant Mechanisms
Source: Molecules. 2025 Aug 6;30(15):3294. doi: 10.3390/molecules30153294 (PMC12348251; doi:10.3390/molecules30153294)
Supplement: Supplementary file 1 [file molecules-30-03294-s001.zip › Table S5.pdf]

Table S5. Combination Index (CI) values representing the interaction effects of EOZ and EOH mixtures on $ABTS^{•+}$ radical scavenging activity.

Formulation Ratio (EOZ:EOH, v/v)	D_1 (EOZ, µg/mL)	D_2 (EOH, µg/mL)	Dx_1 (IC_{50} of EOZ, µg/mL)	Dx_2 (IC_{50} of EOH, µg/mL)	D_1/Dx_1 (EOZ)	D_2/Dx_2 (EOH)	CI	Interaction Effect
1:1	1.50±0.03	1.50±0.03	2.80±0.04	21.50±0.20	0.54±0.01	0.07±.0.001	**0.61±0.03 c**	Synergism
1:2	2.00±0.04	4.00±0.07	2.80±0.04	21.50±0.20	0.71±0.02	0.19±0.004	**0.90±0.06 a**	Slight Synergism/Near-Additive
2:1	2.00±0.04	1.00±0.02	2.80±0.04	21.50±0.20	0.71±0.02	0.05±0.002	**0.76±0.05 b**	Synergism

EOZ and EOH represent essential oils isolated from *Zingiber officinale* (Rosc.) rhizomes and *Humulus lupulus* (L.) strobiles, respectively. Mixtures were prepared at volume ratios of EOZ to EOH (v/v): 1:1, 1:2, and 2:1. CI values (mean ± SD) were calculated based on the median-effect principle using the Chou–Talalay method [33]. D_1 and D_2 denote the concentrations of EOZ and EOH, respectively, in the mixture, required to cause 50% inhibition of $ABTS^{•+}$. Dx_1 and Dx_2 represent the concentrations of EOZ and EOH, respectively, needed to achieve the 50% level of inhibition (IC_{50}), when used individually. Interpretation: CI < 1 indicates synergism, CI ≈ 1 additive effect, and CI > 1 antagonism. Different letters represent statistically significant differences between CI values, as determined by Tukey's test ($p < 0.05$).
