# Supplementary figures and images for: New Insights into the Synergistic Bioactivities of Zingiber officinale (Rosc.) and Humulus lupulus (L.) Essential Oils: Targeting Tyrosinase Inhibition and Antioxidant Mechanisms
Source: Molecules. 2025 Aug 6;30(15):3294. doi: 10.3390/molecules30153294 (PMC12348251; doi:10.3390/molecules30153294)

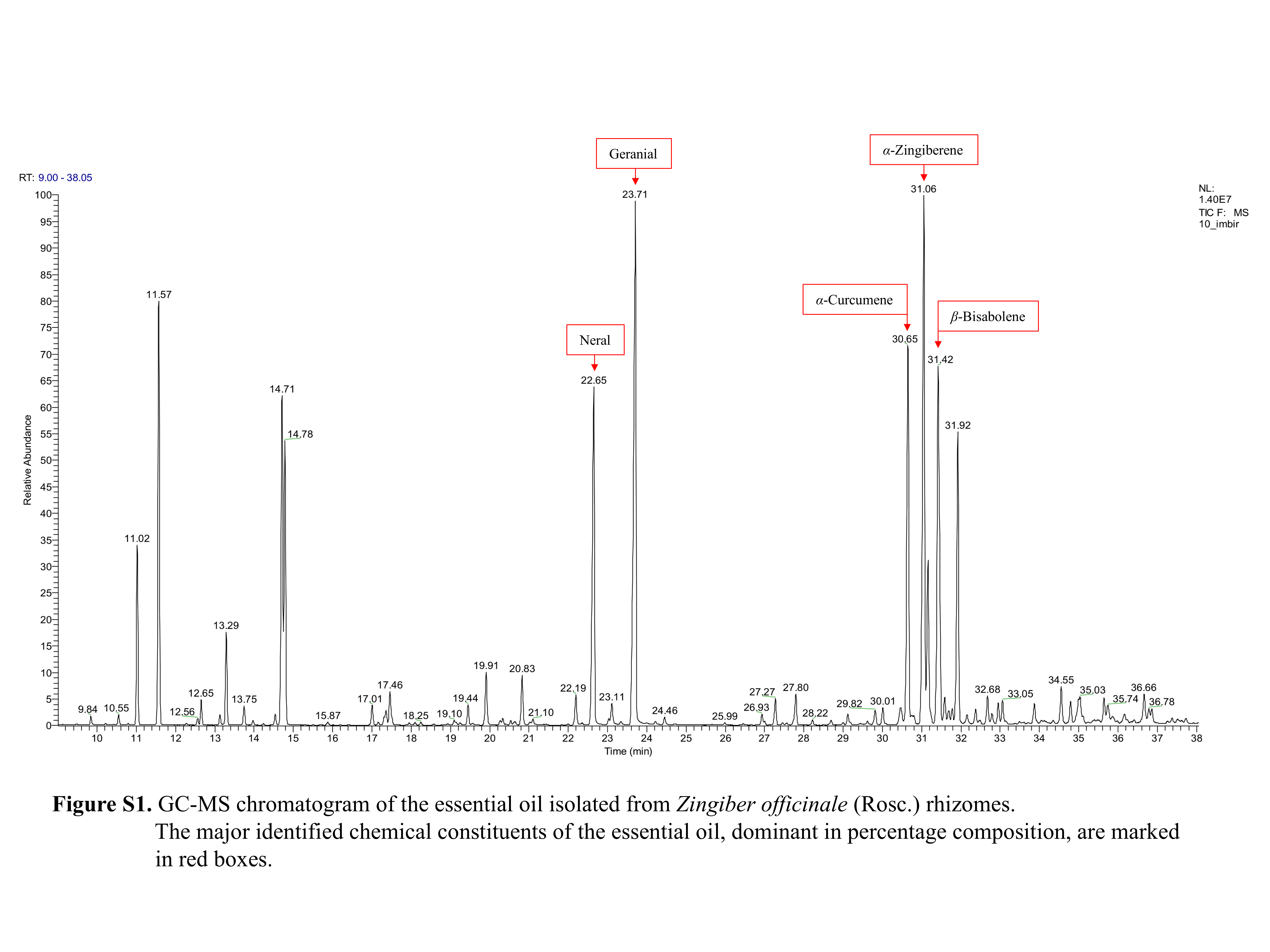

Supplement: Supplementary file 1 [file molecules-30-03294-s001.zip › Figure S1.PNG]

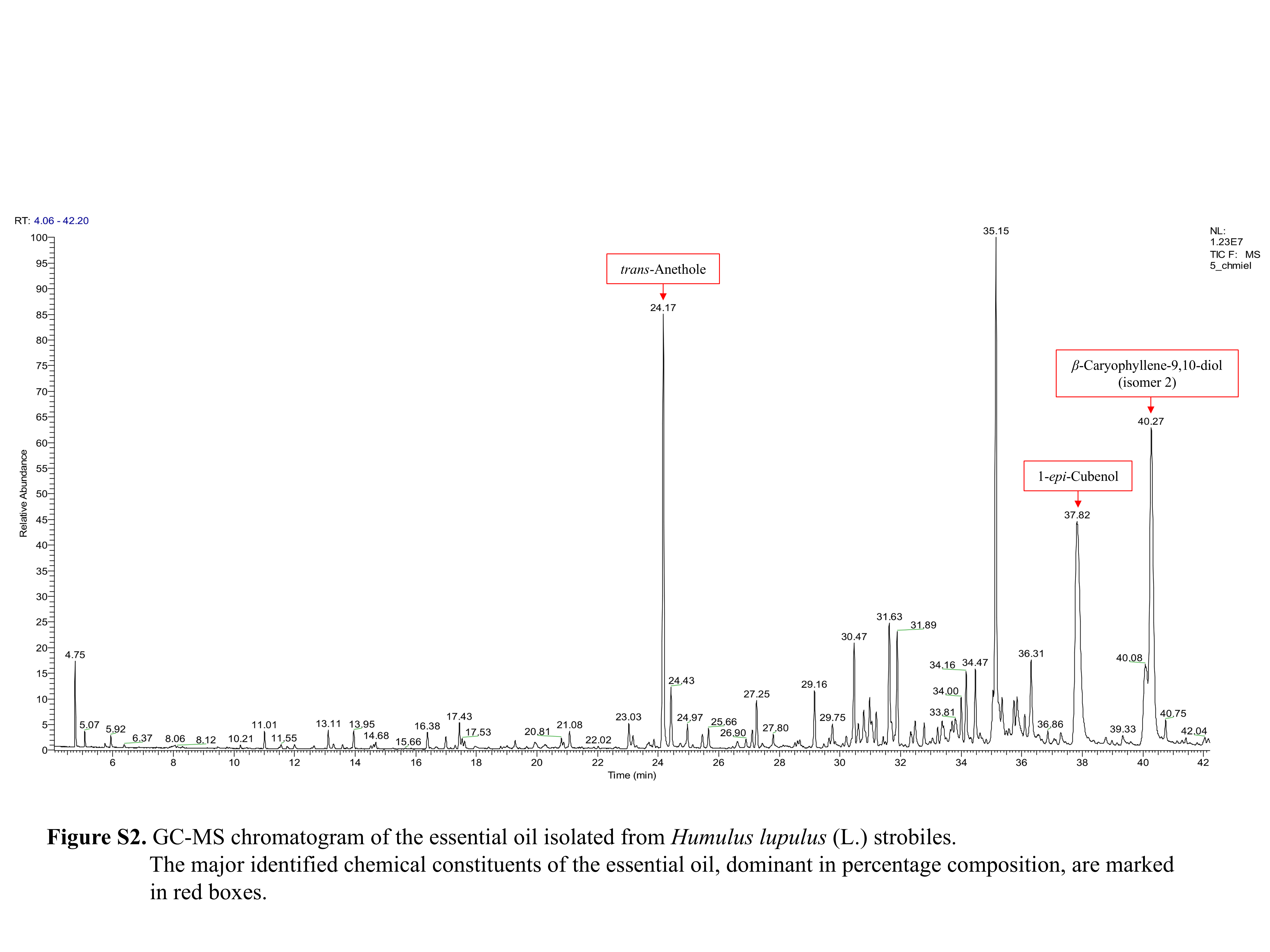

Supplement: Supplementary file 1 [file molecules-30-03294-s001.zip › Figure S2.PNG]
